# Evidence based herbal drug standardization approach in coping with challenges of holistic management of diabetes: a dreadful lifestyle disorder of 21st century

**DOI:** 10.1186/2251-6581-12-35

**Published:** 2013-07-04

**Authors:** Raman Chawla, Pallavi Thakur, Ayush Chowdhry, Sarita Jaiswal, Anamika Sharma, Rajeev Goel, Jyoti Sharma, Smruti Sagar Priyadarshi, Vinod Kumar, Rakesh Kumar Sharma, Rajesh Arora

**Affiliations:** 1 Institute of Nuclear Medicine and Allied Sciences, Brig SK Mazumdar Marg, Delhi, India; 2 Department of Plant Sciences Room 4D70 - 51, Campus Drive College of Agriculture and Bioresources University of Saskatchewan Saskatoon, Saskatchewan, Canada; 3 Office of CC R&D (LS & IC), Defence Research and Development Organisation, DRDO Bhawan, New Delhi, India

**Keywords:** Diabetes, Standardisation, Herbal drugs, Drug development

## Abstract

Plants by virtue of its composition of containing multiple constituents developed during its growth under various environmental stresses providing a plethora of chemical families with medicinal utility. Researchers are exploring this wealth and trying to decode its utility for enhancing health standards of human beings. Diabetes is dreadful lifestyle disorder of 21st century caused due to lack of insulin production or insulin physiological unresponsiveness. The chronic impact of untreated diabetes significantly affects vital organs. The allopathic medicines have five classes of drugs, or otherwise insulin in Type I diabetes, targeting insulin secretion, decreasing effect of glucagon, sensitization of receptors for enhanced glucose uptake etc. In addition, diet management, increased food fiber intake, Resistant Starch intake and routine exercise aid in managing such dangerous metabolic disorder. One of the key factors that limit commercial utility of herbal drugs is standardization. Standardization poses numerous challenges related to marker identification, active principle(s), lack of defined regulations, non-availability of universally acceptable technical standards for testing and implementation of quality control/safety standard (toxicological testing). The present study proposed an integrated herbal drug development & standardization model which is an amalgamation of Classical Approach of Ayurvedic Therapeutics, Reverse Pharmacological Approach based on Observational Therapeutics, Technical Standards for complete product cycle, Chemi-informatics, Herbal Qualitative Structure Activity Relationship and Pharmacophore modeling and, Post-Launch Market Analysis. Further studies are warranted to ensure that an effective herbal drug standardization methodology will be developed, backed by a regulatory standard guide the future research endeavors in more focused manner.

**Electronic supplementary material:**

The online version of this article (doi:10.1186/2251-6581-12-35) contains supplementary material, which is available to authorized users.

## Introduction

Herbal drugs have been used since the inception of human beings on this planet and as a result is almost as old as life itself. In the modern world, the traditional (herbal) medicines assumed a significant proportion of > 83 billion dollars annual production (2008), increasing exponentially [1]. In developing countries, 70-95% of the population relies on herbal medicines for primary care mainly due to cost imperatives or unavailability of conventional drugs. In India, in spite of over 80% of the population dependent upon herbal drugs; it occupies less than 2.5% of the global market share. On the other hand, > 60% market share is being controlled by European Union and North America while 16% being shared by Japan and rest 19% by ASEAN countries [2, 3].

Herbal medicines, containing active ingredients in complex chemical mixtures developed as crude fractions, extracted from aerial or underground parts of plant or other plant material or combination thereof, are widely used in health-care or as dietary supplements. One of the major drawbacks of these medicines is limited bioavailability, being poorly absorbed if taken orally [4–6]. According to an estimate of the World Health Organization (WHO), about 80% of the world population still uses herbs and other traditional medicines for fulfilling their primary health care needs [7]. It is essential to understand that its effectiveness may vary and it might interact with other drugs leading to contraindications. Safety considerations regarding toxicological analysis, pre-clinical and clinical trials are essential prior to adoption of any herbal medicine. At present, herbal formulations have reached widespread acceptability as therapeutic agents for diabetics, arthritics, liver diseases, cough remedies, memory enhancers and adoptogens [8]. In spite of such wide acceptability, the number of *standardized herbal drugs* is less due to lack of regulatory standards and implementation protocols.

Standardization requires a lead/natural plant product to be authenticated at origin itself by adoption of good agricultural practices [9]/ collection strategies from wild and good manufacturing practices for extraction modes and related parameters [10–13]. The acceptance of lead as a future drug candidate requires correct identification, authentication and concentration of active principle [14, 15]/ defined quantities of active components in poly herbal formulations [16, 17]. The regulatory approvals to ascertain consistent chemical profile and biological activity of future drug candidate [18] includes a) quality assurance by determining adulterants, pesticides residue, aflatoxin content, bacterial/fungal growth and heavy metals contamination etc. [9]; b) prevention of adverse reactions by evaluating pharmacodynamics, pharmacokinetics, dosage, stability, self-life and toxicity (acute/ chronic) etc. [19]; c) reproducibility by repetitive testing using different batches to control batch-to-batch variation and development of standard assay markers [17] and; d) chemiinformatic approaches to ensure that pharmacological profiles matches with the activity profiles of active constituents of drug itself.

Diabetes, a dreadful lifestyle metabolic disorder with every 5th Indian as diabetic by 2025 (40 million diabetics in India expected to be 70 million by 2025) [20]. It is a silent epidemic that directly affects glucose catabolism leading to energy yielding changes. The chronic effects include blindness (2%); visual handicap (10%); diabetic retinopathy and neuropathy; sensory loss and damage to limbs [21]. In India, the difference in number of cases being affected in urban population with respect to rural is 8% because of changes in lifestyle and consumption patterns [22]. Globally Type I diabetes, an autoimmune disorder in which beta cells are not functional, affects 10% of diabetic population while 90% cases falls into category of Type II wherein down regulation of receptors leads to insulin non-responsiveness [23]. Type II diabetes produces mild symptoms like fatigue, increased thirst and hunger, weight loss, blurred vision, frequent urination and slow healing of wounds or sores, and can be controlled with a healthy diet, exercise and weight loss. The genetic defects at neonatal level or syndromes causing beta cells destruction leads to various complications such as maturity- onset diabetes of the young (MODY) or neonatal diabetes mellitus (NDM) [23]. There are more than 1000 plants which are used in anti diabetic herbal formulations and among them about 100 plants have been scientifically validated [24]. However, no single approved herbal drug is available till date for mass usage. It is essentially due to lack of standardization methodologies adopted prior to development of drug.

The present review focuses on the herbal standardization models that can be useful for development of evidence based holistic natural plant products with a special case study on ‘management of diabetes’. It provides an in depth analysis of limitations of treatment methodologies; available herbal alternatives; contraindications vs. complications criterion and novel models for standardization of herbal drugs.

### Diabetes: a dreadful lifestyle disorder of 21st century

India is amongst the top most countries followed by China and USA where Diabetes still plagues the society with 32, 26 and 18 million cases respectively [25]. Studies suggest that the average rate of incidence of diabetes in India has reached a value of 7% annually as a result of growing urbanization and changing lifestyle patterns [26]. The average rate at which diabetes causes upsetting loss to the world economy has been figured out to be 12.5% annually [27]. The major causes of diabetes includes either complete absence of insulin hormone (Type I) due to auto-immune disorder/genetic defects/ abnormal physiology (unknown causes) or inadequate biological response towards insulin (Type II) due to down-regulation of receptors (usually in adults due to obesity or other lifestyle factors) [28–30], leading to elevated blood glucose levels [24]. Such elevated levels, if not controlled/managed by external supplementation, leads to energy starvation and deleterious effects on multiple organs like kidney, heart, eyes or nerves and 50% of the diabetic people show such complications at some later stages of chronic metabolic disorder [21, 31].

In Type I diabetes, an increased urge of thirst, constant hunger, weight loss, extreme fatigue, blurred vision and diabetic ketoacidosis may occur while in case of Type II diabetes, frequent urination, persistent hunger, distorted vision, weight loss and extreme exhaustion are primary symptoms. Secondary complications may include a genetic defect of the β cell leading to genetic alterations such as Maturity Onset Diabetes of the Young (MODY) or Neonatal Diabetes mellitus (NDM), consequently making the body incapable of controlling the blood glucose levels [23]. It has been reported that one of the causative factors responsible for the pathophysiology of chronic diabetes includes free radical induced oxidative stress, analogous to radiation-induced acute multi-organ dysfunction syndrome [32, 33]. In both cases, advanced glycation end products (AGEs) [34, 35] are formed by non enzymatic glycosylation of proteins and they tend to accumulate in body tissues, thereby augmenting the process of free radical formation. This consequently results in the progression of the disease into a Multiorgan Dysfunction Syndrome (MODS), thereby posing adverse effects on multiple organs including liver, kidney, pancreas etc. [36]. Antioxidants of either synthetic or herbal origin might be utilized in neutralizing free radicals, thereby mitigating the impact of such diseases.

### Existing approaches for diabetes management

The basic treatment approach is to minimize elevated blood sugar (glucose) level without causing abnormal reduction (hypoglycemia) by using oral antidiabetic drugs; insulin administration; nutritional dietary aid, diet management and scheduled exercise.

WHO reports (1997) showed that between 20%-50% of people with type II diabetes can control their blood glucose levels by dietary modification alone [22]. More than 50% of calorific value of human diet is fulfilled by starch based foods. Starch contains amylose and amylopectin in the ratio of 1:3. Extensive branching in amylopectin and linear long chains of amylose typically resist digestion and hence play a role in resistant starch (RS) formation. Mechanisms and processes by which foods, rich in fiber and resistant starch, exert their disease control properties are not well understood. Some of these factors believed to have effects on rate of digestion and absorption. These include source of food material, its components, physical nature, presence of enzyme inhibitors, anti-nutrients as well as processing methods [37]. The enzymes responsible for carbohydrate hydrolysis are excluded by the fibre components in food thereby slowing down the rate of hydrolysis, impersonating resistant starch metabolism [38]. RS based diet related study on humans depicted increased insulin sensitivity in noninsulin-resistant subjects by changing both adipose tissue and skeletal muscle metabolism can be linked with elevated levels of systemic concentrations of both ghrelin and SCFAs (short chain fatty acids) [39]. High amylose starches have potential of controlling high-fat diet-induced obesity by modulating hepatic fatty acid oxidation [40] while RS induces increase in the levels of anti-diabetic hormones i.e., glucagon-like peptide-1 (GLP-1) and peptide YY (PYY) [41, 42]. Thus, food containing high fiber content as in oat bran, flax, celery etc., low sugar e.g. papaya, cranberries, bitter gourd etc. and high water content e.g. Lettuce, tomato etc. are recommended for diabetes management [43, 44].

The diet alone can reduce glucose burden on the body, thereby preventing excessive utility of anti-diabetic modalities and also delays insulin resistance. However, the physiological response due to glucose homeostasis linked with energy currency attributes, exercise/ physical work in daily schedule is essential to channelize the energy and also to revitalize the cells of the body. The combination of these two lifestyle modifications i.e., exercise and diet can either reduce or delay the incidence of diabetes by over 50% [45].

The definite therapy for diabetes include target based hypoglycemic drugs (by enhancing beta cell stimulation or by reducing the gluconeogenesis etc.) alone or in combination with insulin based on type and progression of ailment over the years. There are five major classes of oral antihyperglycemic drugs and a wide variety of insulin (short acting / long acting) available for comprehensive management of Diabetes. These five distinct classes of oral antihyperglycemic drugs include the Sulfonylureas (SUs), Meglitinides, Biguanides, Thiazolidinediones (TZDs)/glitazone, α-glucosidase inhibitors. These agents fall into two broad categories based on mechanism of action, which are “Secretagogues”, or drugs that augment insulin supply (sulfonylurea, non-sulfonylurea secretagogues, and insulin); and “Sensitizers”, or drugs that assist insulin action (biguanides, α-glucosidase inhibitors, and thiazolidinediones).

**Figure 1:**
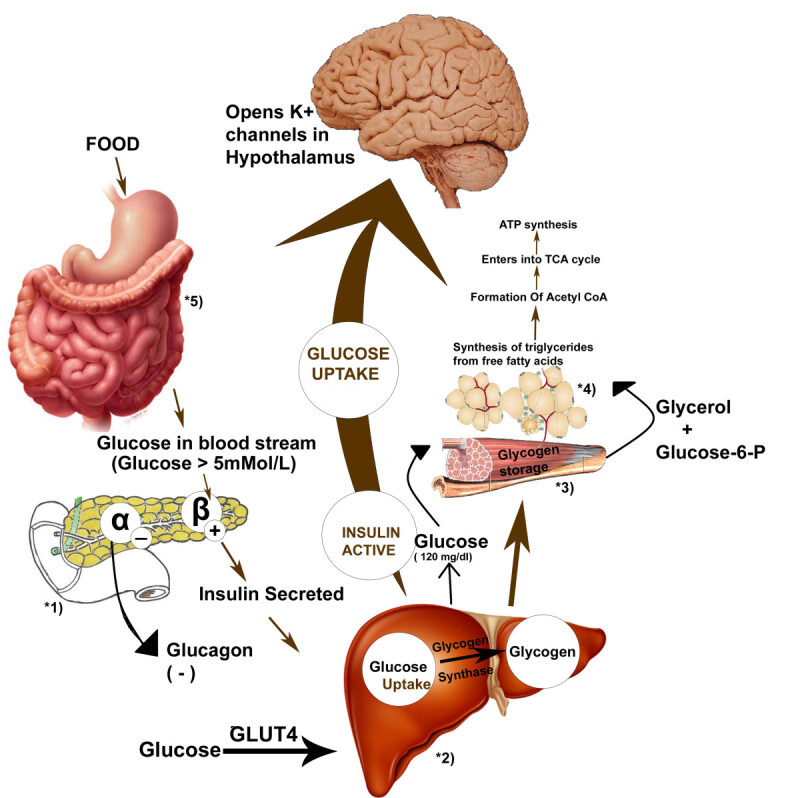
**Various physiological targets of synthetic drugs; (1)-Meglitinides, Sulphonylureas [(Glimiperide (Amaryl); Glipizide (Glucotrol); Glipizide-gits (Glucotrol-XL); Glyburide (Diabeta, Micronase); Glyburide micronized (Glynase); Tolbutamide (Orinase); Chlorpropamide (Diabinese); Tolazamide (Tolinase); Acetoheximide (Dymelor)] & Phenylalanine derivatives; Repaglinide (Prandin) Nateglinide (Starlix), act on pancreas to stimulate insulin secretion by blocking K + ions in β-cells, (2)- Biguanide & Thiazolidine diones act on liver to decrease Gluconeogenesis; (3 & 4)- Biguanide Metformin (Glucophage, Riomet) Metformin-XR (Glucophage XR) act on muscle and adipose tissue to augment peripheral glucose uptake & Thiazolidine diones (Rosiglitazone (Avandia) Pioglitazone (Actos) increase insulin sensitivity** ***via*** **activation of receptors; (5) α-glucosidase inhibitors [(Acarbose (Precose) Miglitol (Glyset)] act on intestine to delay glucose absorption.**

**Figure 2:**
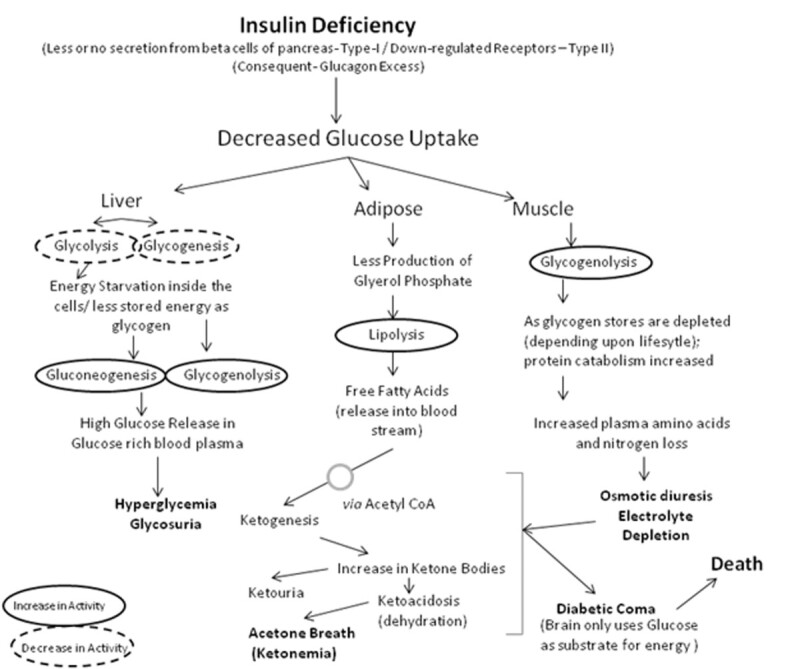
**Metabolism during diabetes.**

The commercially available drugs under these above-mentioned categories, their physiological targets and mode of action are given in Figure 1. Metabolic affects of diabetes caused due to insulin deficiency are given in Figure 2. It has also been reported that activated carbon sphaeroides, by virtue of large specific area, fine porosity, high mechanic strength and excellent adsorption abilities allow intestinal absorption of glucose and thereby helps in equilibrating blood glucose towards the more balanced level [46].

### Complications associated with anti-diabetic modalities

Around 3.2 million deaths (six deaths/minute) every year is attributable to complications of diabetes; [26, 27]. The side effects or complications associated with diabetes pharmacotherapy (synthetic drugs) can be divided into three categories as: a) common side effects; b) uncommon side effects and; c) rare side effects. The most common side effects associated with the oral hypoglycemic drugs include hypoglycemia or extremely low blood sugar (symptoms include profuse sweating, tremor, shakiness, dizziness, hunger, mental confusion, coma, and a rare risk of stroke or death), Gastrointestinal side effects (abdominal pain, nausea, vomiting, diarrhea, gassiness, and bloating), edema (fluid in legs and ankles), increase in “bad” cholesterol (LDL). Certain uncommon side effects include congestive heart failure, Anemia (low red blood cell counts) and allergic reactions. On the other hand, rare side effects/complications include thrombocytopenia (low blood platelet counts), lactic acidosis (build up of acid in the blood), leucopenia (low white blood cell counts), macular edema (eye problems) and liver disease/liver failure [47].

## Herbal alternatives and management of diabetes

The purpose of this article is to examine the effect of herbal medicines in the treatment of diabetes, focusing on potential benefits and risks, and to provide sufficient ways for the proper standardization of herbal drugs. The associated complications of synthetic drugs have lead to a shift towards locating natural resources showing anti diabetic activity. The Indian prehistoric literature reports more than 800 plants with antidiabetic properties while ethno pharmacological surveys indicate that more than 1200 plants can be used for hypoglycemic activity [48].Herbs are also known to provide symptomatic relief and aid in the prevention of the secondary complications of the disease including cholesterol lowering action. Some of these herbs have also been proven to help in the regeneration of β-cells and in overcoming insulin resistance. In addition to maintaining normal blood sugar level, many of these also possess antioxidant activity [24].

Herbal medicines can be broadly classified into the following categories according to their mode of action: a) drugs acting like insulin, b) drugs acting on insulin secreting beta cells, c) drugs modifying glucose utilization, d) drugs showing adrenomimeticism, e) pancreatic beta cell potassium channel blockers, f) cAMP (2nd messenger) stimulators, g) renal glucose resorption inhibitors, , h) herbal drugs providing certain necessary elements like calcium, zinc, magnesium, manganese and copper for the beta-cells, i) drugs regenerating and/or repairing pancreatic beta cells, j) effectors of size and number of cells in the islets of langerhans, k) glycogenesis and hepatic glycolysis stimulators, l) drugs preventing pathological conversion of starch to glucose by inhibition of β –galactosidase, α–glycosidase & alpha-amylase, m) drugs preventing oxidative stress that is possibly involved in pancreatic ß-cell dysfunction found in diabetes [24]; details of the same is given in the Table 1.

**Table 1:** **Herbal plants showing antidiabetic activity**

Botanical name	Local name	Mode of action	Chemical constituents	Marketed antidiabetic product
***Abies pindrow***	Morinda / Rodha	Insulin secretagogue activity	Volatile oil	-
***Abroma augusta***	Devil’s Cotton	Lowering blood sugar	Fixed oil, alkaloid	-
***Acacia arabica***	Babool	Initiate release of insulin	Arabin	Madhumeha
***Achyranthus aspera***	Chirchiri	Decrease blood sugar	-	-
***Agrimony eupatoria***	-	Insulin releasing and insulin like activity	-	-
***Ajauga iva***	Bugle Weed	Decrease plasma glucose level	-	-
***Allium sativum***	Lehsun	Antihyperglycemic and antinociceptive effect	Volatile oil, allin, allicin	-
***Allium cepa***	Pyaaz	Stimulating effects on glucose utilization and antioxidant enzyme	Protein, carbohydrate, vitamin A,B,C, allyl propyldisulphide	-
***Aloe vera***	Gheequar	-	Aloin glycoside	-
***Aloe barbadensis***	Gheequar	Stimulating synthesis and/or release of insulin	Barbaloin, isobarbaloin, resin	-
***Amaranthus spinosus***	Kataili Chaulai	-	-	-
***Anacardium occidentale***	Kaju	-	Flavonols, terpenoids, coumarin, phenolics, essential oil	-
***Andrographis paniculata***	Kalmegh	Increase glucose metabolism	Diterpenoid lactone andrographoloid	Diagon
***Annona squamosa***	Sharifa	Hypoglycemic, antihyperglycemic, Increases plasma insulin level	Acetogenins- squamosin B, squamosamide, reticulatain-2, isosquamosin	-
***Artemisia pallens***	Davana	Hypoglycemic, increases peripheral glucose utilization or inhibits glucose resorption	Essential oil, davanone	-
***Averrhoa bilimbi***	Bilimbi	Increase serum insulin level	-	-
***Azadirachta indica***	Neem	Glycogenolytic effect due to epinephrine action was blocked	Nimbidin, Nimbin, Nimbidol, Nimbosterol	Dianex, Diamed, Madhumeha, Diaveda, Diabeta
***Beta vulgaris***	Chukandar	Reduce blood glucose level by regeneration of β cells	-	-
***Bidens pilosa***	Compositae family	-	Polyacetylenic glucoside	-
***Bixa orellana***	Annotta	Increase plasma insulin conc. & increase insulin binding on insulin receptor	Oleo-resin	-
***Boerhaavia diffusa***	Punarnava	Increase in hexokinase activity, decrease in glucose-6-phosphatase and fructose bis-phosphatase activity, increase plasma insulin	Alkaloid punarnavaine, punarnavoside	-
***Brassica juncea***	Rai	Food adjuvants for diabetic patients	Isothiocyanate glycoside singrin, protein, fixed oil	-
***Caesalpinia bonducella***	Karanju	Free radicle scavenging	Fatty oil	-
***Camellia sinensis***	Green Tea (Chai)	Increase insulin secretion	Polyphenolic constituents	-
***Capparis decidua***	Karer	Hypoglycemic, antioxidant, hypolipidaemic	-	-
***Capsicum frutescens***	Mirch	Increase insulin secretion & reduction of insulin binding on the insulin receptor	Capsaicin, pritein	-
***Carum carvi***	Shia Jira	-	V.oil, resin, carvone, fixed oil	-
***Cassia alata***	Ringworm Senna	-	-	Aavirai kudineer, Madhumeha
***Cassia auriculata***	Tarwar	Increase utilization of glucose through increase glycolysis	-	Hyponidd, Mersina, Dianex, Diamed, Aavirai kudineer, Madhumeha, Diasulin
***Catharanthus roseus***	Sadabahar	Increase mobilization of glucose	Indole alkaloid, vincristine vinblastin	Diabeta, Tincture of panchparna
***Cinnamomum zeylanicum***	Dalchini	Elevation in plasma insulin	Volatile oil, tannin, mannitol, calcium oxalate,	Glucoessential capsules
***Clausena anisata***	Rutaceae Family	Stimulate secretion of Insulin	-	-
***Coriandrum sativum***	Dhania	-	Volatile oil, fixed oil, protein	-
***Coscinium fenestratum***	Jharhaldi	Increase enzymatic antioxidants	Barberine, glycoside, saponin	-
***Croton cajucara***	Jamalgota	-	Fixed oil	-
***Cryptolepis sanguinolenta***	Anantmul	Increase glucose uptake by 3T3-L1 cells	Cryptolepine	-
***Eclipta alba***	Bhringraj	Decrease activity of glucose-6-phosphatase & fructose-1-6, bisphasphatase	Ecliptin alkaloid	-
***Embellica officinalis***	Amla	Reduce 5-hydroxymethylfurfural,creatinine albumin level	Vitamin C, tannin	Hyponidd, Diasulin, Diaveda, DWN-12
***Enicostemma littorale***	Chhota Chirayata	Decrease glycosylated Hb & glucose 6 phosphatase	Swertiamarine glycoside	Hyponidd, Glucolib, Glucomap, Glucova
***Eugenia jambolana***	Jamun	Lowers plasma glucose level	-	Glucomap, Glucova, Hyponidd, Dianex, Glucolib, Aavirai kudineer, Madhumeha, Diagon
***Eucalyptus***	Neel giri	Increase insulin secretion from clonal pancreatic beta line (BRIN-BD 11)	Essential oil, cineol	-
***Euphrasia officinale***	Eyebright	-	-	-
***Ficus religiosa***	Peepal	Initiating release of insulin	Tannin	-
***Ficus bengalensis***	Bargad	Rising serum insulin	Tannin	-
***Ficus carica***	Anjir	-	-	-
***Gymnema montanum***	-	Antioxidant & Antiperoxidative	-	-
***Gymnema sylvestre***	Gudmar	Lowers plasma glucose level	Gymnemic acid, quercital	Tincture of panchparna, Pancreas tonic, Gurmar powder, Diaveda, Glucolib, Glucocare, Hyponidd, Mersina, Dianex, Madhumeha, Diagon, Glucoessentials, Diasulin
***Gentiana Olivier***	-	Lowers plasma glucose level	Iso-Orientin C-Glycoside	-
***Glycerrhiza glabra***	Mulethi	-	Triterpenoid, saponin, glycerrhizin	Glucocare
***Gynura procumbens***	-	Lowers plasma glucose level	-	-
***Hibiscus rosa sinensis***	Gudhal (China Rose)	Stimulate Insulin secretion from Beta cells	Vit. B, C, Fat	-
***Helicteres isora***	Indian Screw Tree	Decrease plasma triglyceride level & insulin sensitizing activity	Saponin, tannin, lignin	-
***Hordeum vulgare***	Jau	-	-	-
***Hovenia dulcis***	-	-	Flavonoids	-
***Ipomoea aquatica***	Kalmisag	-	Carotene	-
***Ipomoea batata***	Shakarkand	Reduce fasting blood sugar level & serum glucose level	-	-
***Juniperus communis***	Hauber	Increase Peripheral Glucose Consumption & Induce Insulin Secretion	-	-
***Lupinus albus***	Turmas	Lower Serum glucose level	Alkaloid, fatty oil, asparagines	-
***Luffa aegyptiaca***	Ghiatori	Lactigogue activity	Fatty oil	-
***Leucas lavandulaefolia***	Kumbha	Reduce Blood Glucose Level	-	-
***Lagerstronemia speciosa***	Jarul	-	-	-
***Lepidium sativum***	Halim, Hurf	-	-	-
***Mangifera indica***	Mango	Reduction of intestinal absorption of glucose	Mangiferin	-
***Myrtus communis***	Vilayati Mendhi	Lower blood glucose level	V.oil mirtii oleum	-
***Memecylon umbellatum***	Anjani	Lower serum glucose	-	-
***Momordica cymbalania***	Kadavanchi	Reduce blood glucose level	-	-
***Mucuna pruriens***	Kiwach	Reduce blood glucose level	-	-
***Musa sapientum***	Banana	Reduce blood glucose & glycosylated Hb	-	-
***Momordica charantia***	Karela	Reduce blood glucose level	Momordicine alkaloid, ascorbic acid	Hyponidd, Mersina, Dianex, Diamed, Madhumeha, Glucoessentials, Diasulin, Glucolib, Glucocare, Diabeta, Glucomap, Pancreas tonic, Tincture of panchparna
***Morus indica***	-	Increase glucose uptake	-	-
***Murraya koeingii***	Curry Leaf	Increase glycogenesis, decrease glycogenolysis & gluconeogenesis	-	Madhumeha
***Nelumbo nucifera***	Lotus	Reduce blood sugar level	Nuciferin, nornuciferin	-
***Ocimum sanctum***	Tulsi	Reduce blood sugar level	V.oil, phenol, aldehyde, fixed oil, alkaloid, tannin, ascorbic acid	Glucoessential capsules
***Olea europia***	Olive	Potentiation of glucose, induced insulin released and increase peripheral uptake of glucose	Oleuropeoside	-
***Opuntia Ficus indica***	Indian Fig	-	-	-
***Pandanus odorus***	Kevra	Decrease plasma glucose level	Essential oil	-
***Panax ginseng***	Pannag	Lowering blood sugar level	Glycans, panaxans I,J,K & L	Glucoessential capsules
***Punica granatum***	Anar	Lowering blood sugar level	Vitamin C, protein, tannin, gallic acid, pelletierine	-
***Picrorrhiza kurroa***	Katuka	Decrease serum glucose	Picrorrhizin, kutkin	-
***Phyllanthus amarus***	Bhui Amla	Lowering blood sugar level	Alkaloids	Mersina
***Phaseolus vulgaris***	Lobia	Hypoglycemic, hypolipidemic, inhibit alpha amylase activity, antioxidant	-	-
***Salacia oblonga***	Chundan	Inhibition of alpha glucosidase activity	-	Aavirai kudineer
***Salacia reticulata***	Anukudu Chettu	Inhibition of alpha glucosidase activity	-	-
***Swertia chirayata***	Chirayata	Stimulates insulin release from islets	Zanthone mangiferin, gentianine, swerchirin	Hyponidd
***Syzygium cumini***	Jamun	Decreases blood glucose level	-	Mersina, Diasulin, Diaveda, Pancreas tonic, DWN-12, Diabeta
***Scoparia dulcis***	Mithi Patti	Decrease glycosylated Hb, Insulin secretagogue activity	-	Diasulin
***Trigonella foenum graceum***	Methi	Decrease blood glucose concentration	Protein, fat, V. oil, fixed oil, carbohydrate	Mersina, Glucoessesntials, Diasulin, Glucolib, Diaveda, Syndrex
***Tribulus terrestris***	Gokhru	Decrease serum glucose	Harmine	Diaveda
***Tinospora crispa***	Giloe	Anti-hyperglycemic, stimulates insulin release from islets	-	-
***Tinospora cardifolia***	Giloe	Decrease blood glucose and brain lipid	Berberine, starch	Hyponidd, Mersina, Diagon, Diasulin, Diaveda, Glucova, Diabeta, Pancreas tonic
***Tamarindus indica***	Imli	-	-	-
***Teramnus labialis***	Mashoni	-	Caumarin - fraxidin	-
***Urtifca dioica***	Bichhu Booti	Increase Insulin secretion	Fatty oil	-
***Viscum album***	Vadank	Alpha glucosidase inhibitor	-	Glucoessential capsules
***Vinca rosea***	Sadabahar	Beta cell rejuvenation, regeneration & stimulation	Vincristine, vinblastine	Diabeta, Tincture of panchparna
***Withania somnifera***	Ashwagandha	Decrease blood sugar level	Withanine, somnine, withaferine, withanolides	Dianex
***Xanthium strumarium***	Chhota Gokhru	Increase glucose utilization	Phenolics, caffeic acid	-
***Zingiber officinale***	Adrak	Increase Insulin level & decrease fasting glucose level	Sesquiterpene	Diaveda
***Zizyphus sativa***	Pitni-Ber	Dose dependent reduction in blood glucose level	Tannin	Madhumeha

**Figure 3:**
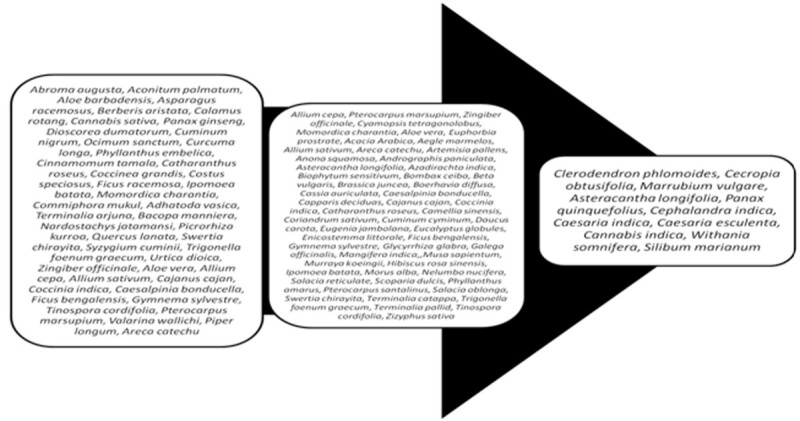
**Advancement in the field of anti-diabetic potential herbals (Extreme left: Traditional Herbs; Middle: Scientifically Validated, yet not clinically proven; Extreme Right: Under clinical trials).**

The plant constituents under the category of polysaccharides, peptides, alkaloids, glycopeptides, triterpenoids, amino acids, steroids, xanthones, flavonoids, lipids, phenolics, coumarins, iridoids, alkyl disulphides, inorganic ions and guanidines are reported to have antidiabetic activity [48]. Alkaloids inhibit alpha-glucosidase and decrease glucose transport through the intestinal epithelium while imidazoline compounds and ferulic acid simulates insulin secretion in a glucose-dependent manner. Polysaccharides, saponin, Triterpenoids, steroidal glycosides cause increase in the levels of serum insulin, reduction in the blood glucose levels and improved tolerance of glucose. In contrast flavonoids suppress glucose level, reduce plasma cholesterol and triglycerides thereby, increasing hepatic glucokinase activity by virtue of enhanced insulin release from pancreatic islets [48]. The vast variety of antidiabetic herbal plants can be classified as accepted/scientifically validated herbal plants, traditional herbal plants, antidiabetic plants that are under clinical trials, as elucidated in Figure 3.

## Contraindications of herbal alternatives

Herbal drugs are prescribed widely because of their effectiveness, less side effects, broad range of action and relatively low cost. However, the non trial drugs are usually not evaluated for purity and consistency of active compounds; they often contain contaminants and might show batch-to-batch variations [49]. The exact mechanism of action in lowering blood sugar is often not known. In addition, these herbs may not work well for everyone and their overall effect may vary in individuals, due to lack of standardization. Side effects can also be patient specific and a combination of these herbs may be required to obtain the desired effect which leads to the development of pre-clinical trials for poly-herbal formulations. The various shortcomings/contraindications of herbal medicines includes: a) Being self prescribed, quality assurance is not guaranteed and also may interact with other drugs, b) contraindications of herbal drugs and associated unusual beliefs, c) contain powerful, pharmacologically active compounds that needs to be evaluated for drug-drug interactions, d) usually cause hepatic and renal problems if taken in excess; due to lack of pharmacodynamics and pharmacokinetics data and slow clearance rate from the body, e) difficulty in identification of the causative agent associated with the adverse reactions encountered as these often contain multiple ingredients, f) lack of standardization while the formulation of herbal drug i.e. delivery system for particular target is generally not validated, g) mode of action of herbal plant constituents is not clear enough to support therapeutic utility.

Complications refer to the changes or problems that occur when treatment goes beyond the desired effect and leads to unwanted symptoms. They can be mild or severe varying from person to person. Usually complications/side effects arise in the case of chemical or synthetic drugs. Herbal plants do not usually show any adverse reactions, if tried and tested over a larger period of human era. Whereas due to lack of standardization, pharmacological and toxicological evaluation, certain undesired effects might develop as a result of intake of non standardized herbal formulations. Such effects are generally due to the presence of contaminants/adulterants rather than the active constituent itself. These contraindications develop as a physical condition that put some people at risk of danger from using a particular herbal formulation and results in ironical situation of benefit vs. risk [9].

Safety of herbal medicines is of major concern, whether being self-prescribed or used to treat minor and chronic conditions. However, most patients consuming herbal preparations are not aware of the potential adverse effects these preparations [49]. It is essential to ascertain that certain groups of the population especially pregnant and nursing women should be extra cautious as they are more susceptible to herbal adverse reactions or toxicities. Some compounds in herbs can cross the placenta and are clearly linked to birth defects or other problems in newborns. Thus, the safety against teratogenic effects should also be evaluated during pre clinical studies. Children and infants are much more sensitive than adults to the effects of all medicines including herbs [50, 51]. The elderly with cardiovascular problems, diabetes and other chronic diseases may show exaggerated toxic/adverse reactions to herbs. The key to such associated limitations of herbal drugs is to standardize them prior to use commercially.

## Standardization approaches for herbal drugs

Standardization and quality control of herbals is a process involving monitoring of the entire process of bioprosception of natural flora, collection, extraction, bio-activity guided fractionation and formation of herbal drugs [1, 22] utilizing existing technical standards [52]. Standardization of herbal medicines is the process of prescribing a set of standards or inherent characteristics, constant parameters, definitive qualitative and quantitative values that carry an assurance of quality, efficacy, safety and reproducibility [53, 54]. The quality of herbal drugs is affected by numerous factors: a) Mixtures of many constituents that make physiological responses complex yet holistic; b) Active principle (s) are generally unknown; c) Non-availability of selective analytical methods or standard reference compounds limits development of standard chemical fingerprint, required to ascertain efficacy among various batches; d) Natural variability associated with plants both in wild & non-wild varieties; e) Differences in spectra of bioactivity in natural vs. chemo-varieties and chemo cultivars and; f) Variability in source and quality of the raw material etc.

The methods used in harvesting, drying, storage, transportation, and processing (for example, mode of extraction and polarity of the extracting solvent, instability of constituents, etc.) also affect significantly the finished herbal drugs. Unknown compositions and site of targets, batch-to-batch variations and non-standardized methods of development of herbal drugs restricts its commercial utility [8]. All these factors influence the bio-efficacy and reproducible therapeutic effect of herbal drugs. The scientifically validated drugs for *diabetes mellitus* (Figure 3) require adoption of proper standardization methodologies to ensure its efficacy for human use.

### Proposed models in meeting challenges of standardization process

The traditional medicine were prescribed by *rishis* since vedic times specific to a patient or a group of patients based on locally available flora. Accordingly, this science has evolved in many centuries to the present age of ayurveda, unani, siddha etc. The present day bulk requirement of drug while covering many ethnic groups of global population poses a great challenge towards *standardizing* ’*standardisation methodologies*’. It requires state-of-art technologies to ensure that finished product is safe to use and reproducible to ensure adequate supply. One of the accepted approaches is to ascertain specified amount of active ingredient- for e.g., 30-80% of silymarin or silybin in Milk thistle (*Silybum mariannum*) or 30-70% of kavalctones in Kava (*Piper methysticum*) etc.

#### Challenges in identifying ‘marker compounds’ and ‘active principle(s)’

The major challenge is that ‘active principle’ is not necessarily always a ‘marker’ compound. Markers are often chosen to identify correct species as in *Echinacea angustifolia* could be represented by echinacoside; Parthenolide for Feverfee, *Tanacetum parthenium* represents its particular chemotype. Ferulic acid is used as a stability marker to ascertain storage conditions. Fractionation defines the process to reach towards peak of one or two compounds referred as marker and if taken isolated compound, it has been observed that there is a substantial loss of activity and increased toxicity. If there is one compound as standardized marker, the extract could be spiked with pure chemical to ascertain ‘false’ clearance from solving the issue of ‘batch-to-batch’ variation. Thus, various constituents of an extract should be represented by (a) Active principle (s); (b) Active marker (s); (c) Analytical marker (s) and; (d) Negative marker(s). However, it is not possible that in all standardization processes, all such markers are available. In addition, these markers/active principle (s) based standardization itself, creates confusion to ascertain a particular bioactivity profile. It can be understood by the fact that most of natural plant products are standardized by active markers, contributing to efficacy, though clinical efficacy yet to be correlated while rest of commercially available herbal drugs are having analytical markers with defined range of a particular constituent and negative markers are used to screen toxicological aspects. However, the analytical markers represented as ‘total alkaloids content’ provide another mislead as it might be representing a complete set of constituents rather than ‘specific’ constituent itself. The marker content also varies in different seasons of growth, sites of growth and other environmental factors. Any variation is storage or processing method will also cause shift in levels of markers. In addition, at industrial level, raw material is mixed leading to ‘average marker content’ rather than absolute marker level as being standardized in laboratory with controlled amount of raw material. Thus, it is essential to standardization of ‘average marker content’ is absolute necessity prior to shifting to field level production. Normalisation of data is essential to adjust the concentration of marker component with respect to various recipients being added or altering the extraction ratio. Extraction ratio refers to the weight of the plant used as raw material with respect to the yield generated. Fractionation will generate high strength extracts refers to extraction using more selective and less polar solvents while the remaining extract residue will become standardized extract. Thus, it is important to understand that a particular process required to be defined with a finite point wherein the process should be stopped as further fractionation is causing loss of activity. *At present*, *there are no methodologies that can correlate* ‘*activity profile*’ *vs*. ‘*constituent*’*s profile*’ *as being expected from herbal drugs to act*[55–58].

#### Challenges in developing technical standards for standardization process

It is essential to employ different sets of technical standards for proposed standarisation models. The important standards include Pharmacopeial Quality Standards; Marker based on Phytochemical Assays and bioactivity guided fractionation; Process Control Standards; Storage Standards; Poly herbal reference standards and Chemi-informatic approaches based structural standards that have been given in Table 2.

**Table 2:** **Existing**/ **Proposed standards for herbal drug standardisation**

Type	Tests/Applications/Standards used
**Pharmacopeia standards**	Microscopic examination; Authenticity; Quality; Extractive Value; Foreign Matter; Microbial count; Pesticide Residue; Density; Specific Gravity; Loss on drying; Melting point; Viscosity; Refractive Index; Heavy metals
**Marker based phytochemical assays**	Active principle(s), Storage marker(s), Chemical family representing a particular species, chemotype, Solvent System Standardisation, Pharmacopeia standards with respect to reference compound commercially available/ synthetically viable; Chromatographic, IR, MS, UV, NMR based chemical profile marking
**Process control markers**	Batch analysis intermediate testing, Identification tests, Process induced toxicity/ impurity- quality check procedural standards, Start and Close time point average run sample marker(s)
**Storage standards**	Storage standard marker for raw material (conditions standardization); specimen collection and authentication; In process storage of stable intermediate metabolites from fractions (remained stable till finished products); Storage standards for processed material and stability, shelf life and container characteristics, labeling standards for finished product
**Poly herbal reference standards**	Component based single constituent reference standard exclusive and retained its profile in poly herbal formulations, Dietary standards for nutritional aid as supplement, Therapeutic standards for efficacy of composite formulation
**Chemi**-**informatics approaches based structural standards**	Activity descriptors and its correlation modeling with poly-constituent profile based indicators; Pharmacophore standards using Herbal QSAR approaches to identify structural requirements

#### Meeting challenges by employing quality control measures

Quality control measures include right from the proper identification of plants, season and area of collection as well as their extraction and purification processes. Some of the important tests conducted for ensuring quality process include.

##### Macro and microscopic examination

The examination is conducted to ascertain taxonomical classification of source (wild/agriculture) and part of plant being to target so that defined search of adulterants based on a levels of exposure to environment can be evaluated. The plant part under consideration required to be standardized with respect to its variable factors for selection e.g. if leaf is selected as target plant part, leaf constant based on the palisade ratio, vein islet number, vein termination, stomatal number, stomatal index and type of trichomes and stomata should be evaluated [22].

##### Removal of foreign organic matter

This involves removal of foreign organic matter other than source plant to get the drug in pure form.

##### Ash values

Thermo gravimetric analysis (TGA), differential thermal analysis (DTA) and differential scanning calorimetry (DSC) provides information about physical or chemical changes [59–61] and it also provides estimates of total ash, sulphated ash, water soluble ash and acid insoluble ash etc.

##### Moisture content

Moisture content is required to be evaluated precisely to determine actual weight of drug material. Low moisture suggests better stability against degradation of product.

##### Extractive values

Several techniques are being used in extraction of phytochemicals. These include supercritical fluid extraction [62, 63], microwave assisted extraction [64–66], and solid phase extraction [67, 68]. These are indicative weights of the extractable chemical constituents of crude drug under different solvent environment.

##### Qualitative chemical evaluation

It includes identification and characterization of crude drug with respect to phytochemical constituent like phenolics, alkaloids, flavonoids, dietary fiber etc. Phytochemical screening techniques involve botanical identification, extraction with suitable solvents, purification, and characterization of the active constituents of pharmaceutical importance [69]. Differential pulse polarography (DPP) can also be used to study trace amounts of chemicals with detection limits on the order of 10^-8^ M. A DPP method is available for the determination of total hypericin in phytotherapeutic preparations (drops, tablets and capsules) in various buffer systems over the pH range 3.5–10.0 [70].

##### Chromatographic examination

Characterization of the different chemicals on the basis of their interaction with the mobile phase using preparative high performance liquid chromatography (HPLC)- low pressure HPLC (typically under 5 bar) and high pressure HPLC (pressure >20 bar). Liquid chromatography- mass spectroscopy (LCMS) has become method of choice in many stages of drug development as it provides direct chemical fingerprint in correlation with desired bioactivity. Chemical standardization of an aqueous extract of the mixture of the 20 herbs provided 20 chemical compounds serving as reference markers using LC-MS [71, 72]. Such standards will be used to develop poly-herbal formulations. Gas chromatography (GC) and gas chromatography-mass spectroscopy (GC-MS) based headspace solid-phase micro-extraction method with gradient temperature was reported for analysis of the volatile compound [73, 74].

##### Quantitative chemical evaluation

Analysis of the amount of the major classes of active constituents. X-ray powder diffractometry (XRD) is used to analyse different minerals, crystalline materials and metallic based herbal formulations. The tin based herbal drug Vanga Parpam was estimated by XRD and the intense sharp diffraction peaks clearly confirmed the presence of high crystallinity in Vanga Parpam [75]. X-ray powder Diffraction data confirmed the formation of phospholipid complex with emodin [76] and naringenin [77].

##### Toxicological studies

It reveals level of pesticide residues and potentially toxic elements. Safety studies in animals generate LD_50_ value and other applied pre-clinical toxicity results as per the requirements of drug regulations. HPTLC technique is widely employed in pharmaceutical industry in process development, identification and detection of adulterants in herbal product and helps in identification of pesticide content, mycotoxins as well as quality control of herbs and health foods [78, 79].

##### Microbial assay

To establish the absence or presence of potentially harmful microorganisms. It often includes total viable content, total mold and enterobacterial counts. Certain minimum standards are set by different monitoring agencies [80, 81].

### Integrated herbal drug development model

**Figure 4:**
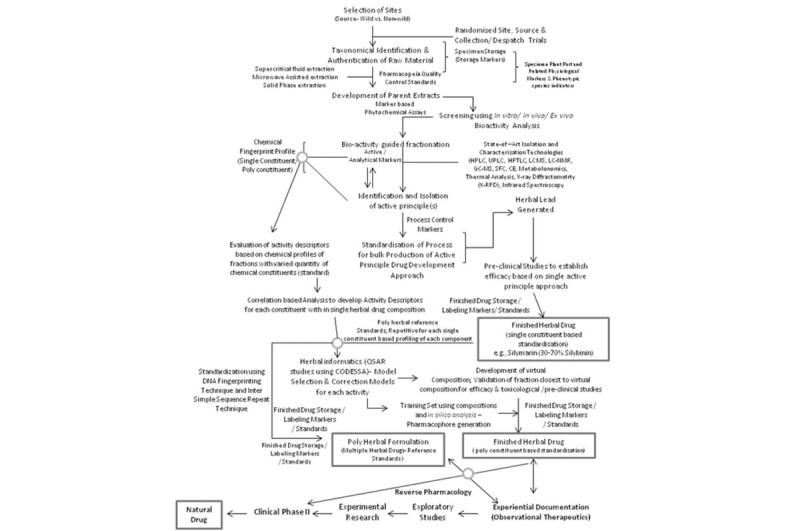
**Integrated herbal drug development standardisation model [Single Constituent based Herbal Medicine; Poly constituent Herbal Drug (based on Chemiinformatics Approaches); Poly herbal formulation; Herbal Drug Development using Reverse Pharmacology].**

India, with pluralistic health care system, having extensive expertise in modern medicine, Indian systems of medicine, and life & pharmaceutical sciences with an approach towards observational therapeutics is an antecedent path towards reverse pharmacology in natural drug development as being proposed [5]. The chemi-informatics based drug development approaches required to be integrated with existing Reverse Pharmacological Approach utilizing above-mentioned technical standards at each stage of process. The classical approach of Bioactivity guided fractionation/identification of leads required to be amalgamated with these modern approaches of natural drug development. Based on this study, we hereby propose an integrated model of herbal drug development model using a) Classical Approach of Ayurvedic Therapeutics (Active principle guided approach); b) Reverse Pharmacological Approach based on Observational Therapeutics; c) Technical Standards for complete product cycle; d) Chemi-informatics, Herbal Qualitative Structure Activity Relationship (QSAR) and Pharmacophore modeling; e) Post Market Analysis (Figure 4). This model could be used to convert the herbal products identified in scientifically validated category (Figure 3) to herbal drugs for holistic management of diabetes.

## Conclusions

Herbal drug standardization is a dynamic phenomenon requires inputs from various branches of life sciences including botanists, plant physiologists, pharmacology, pharmacogonasy, chemiinformatics, biochemistry, toxicology, biotechnology, drug development, natural medicine (Ayurveda, Unani, Siddha etc.) and industrial economic/regulatory affairs. An in depth analysis of each step/stage during natural drug development is necessary to ascertain quality, safety and reproducibility. The complexity of chronic diabetes or lack of awareness leads to sudden onset of diabetes poses a significant risk of occurrence of ketoacidosis and diabetic coma, if untreated/unnoticed respectively. The multi-organ dysfunction syndrome arises through this metabolic disorder can be mitigated/delayed by utilizing holistic approach of herbal drugs, only if these drugs (scientifically validated) undergo a critical standardization process. The proposed model is a key to meet some of the probable challenges of this arena. Further studies are warranted to ensure that an effective herbal drug standardization methodology will be developed, backed by a regulatory standard guide the future research endeavors in more focused manner.

## Authors’ original submitted files for images

Below are the links to the authors’ original submitted files for images. Authors’ original file for figure 1 Authors’ original file for figure 2 Authors’ original file for figure 3 Authors’ original file for figure 4
