# Supplementary figures and images for: Evidence based herbal drug standardization approach in coping with challenges of holistic management of diabetes: a dreadful lifestyle disorder of 21st century
Source: J Diabetes Metab Disord. 2013 Jul 4;12:35. doi: 10.1186/2251-6581-12-35 (PMC7983574; doi:10.1186/2251-6581-12-35)

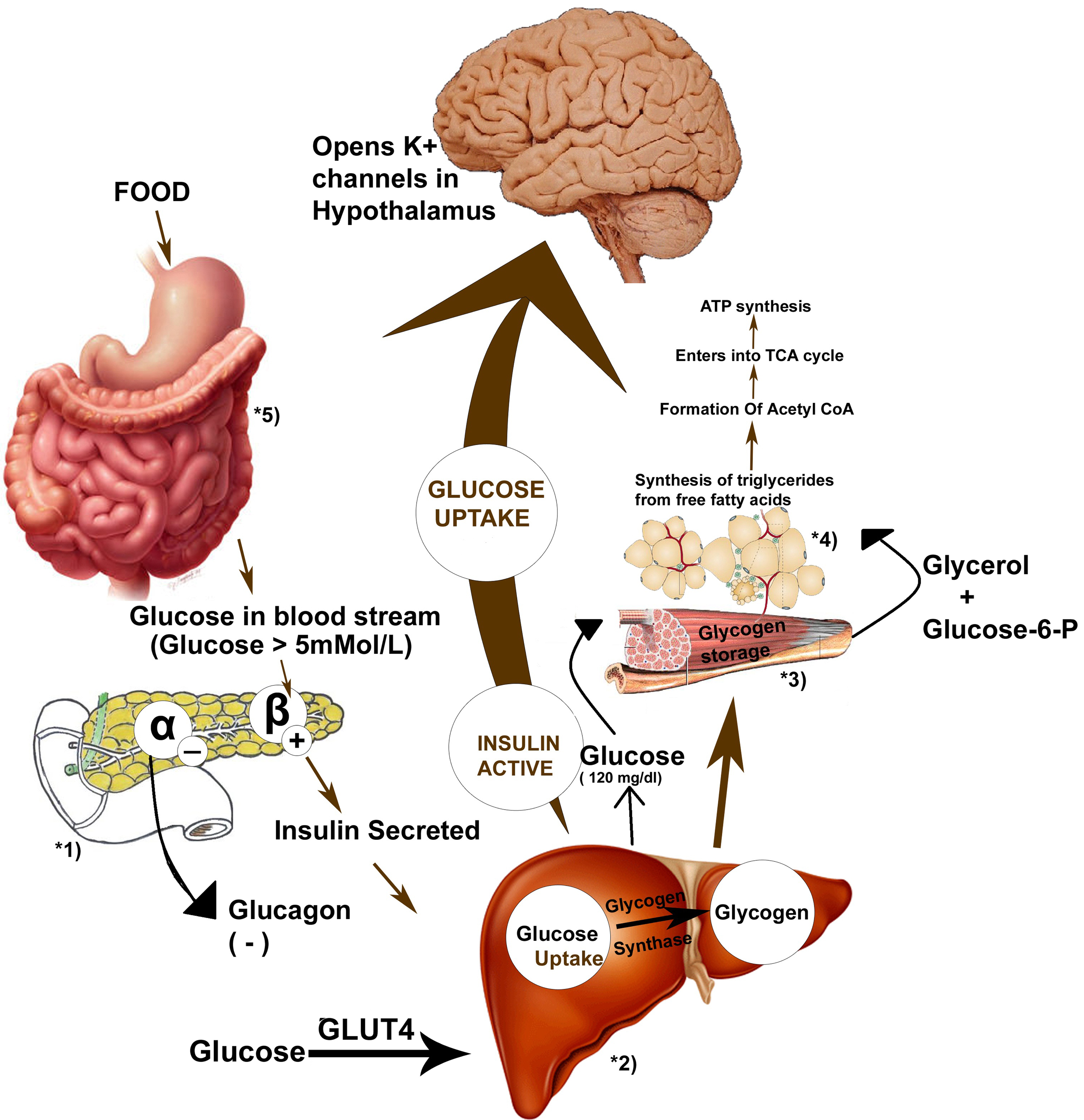

Supplement: Supplementary file 1 — Authors’ original file for figure 1 [file 40200_2013_71_MOESM1_ESM.tiff]

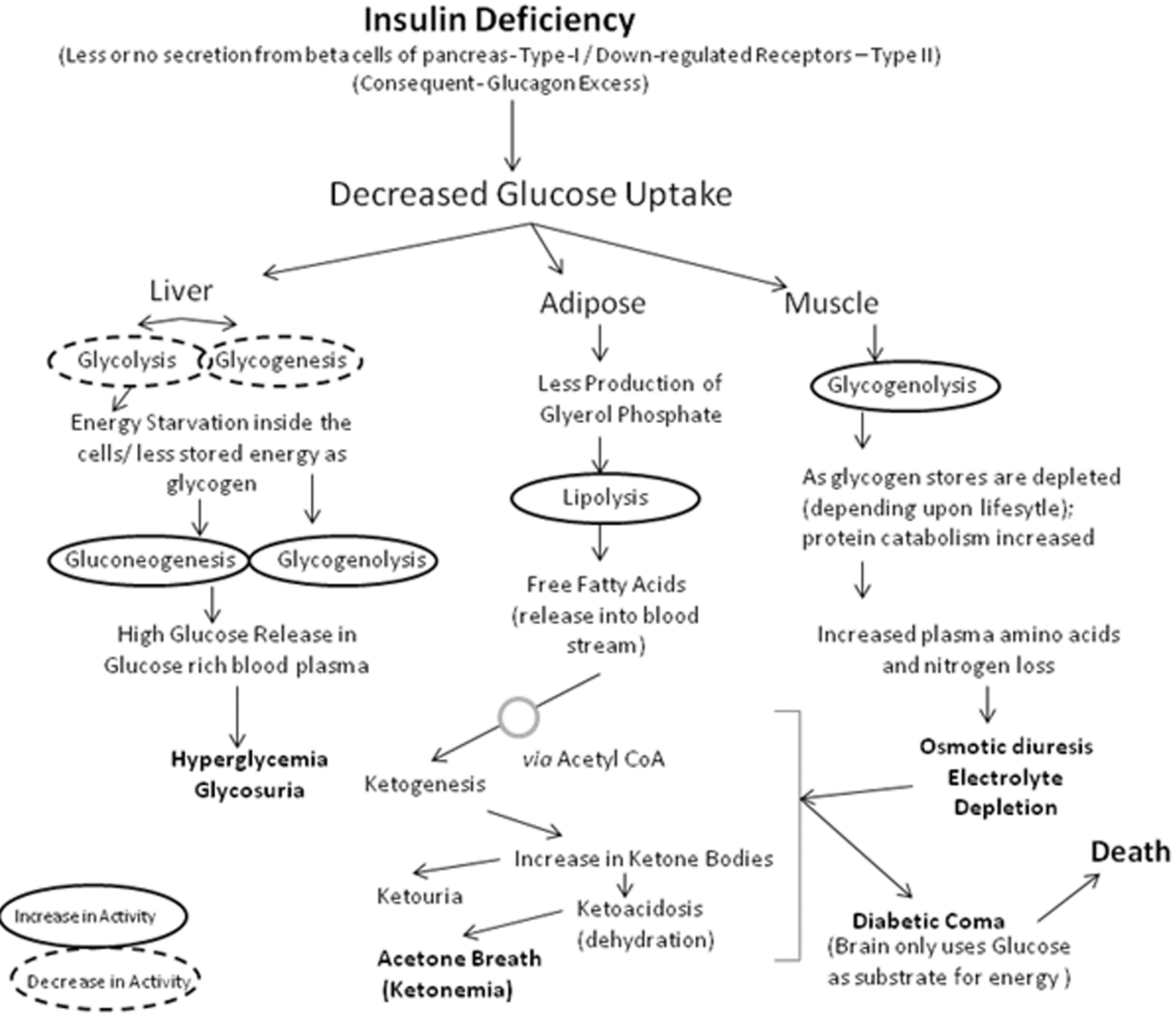

Supplement: Supplementary file 2 — Authors’ original file for figure 2 [file 40200_2013_71_MOESM2_ESM.tiff]

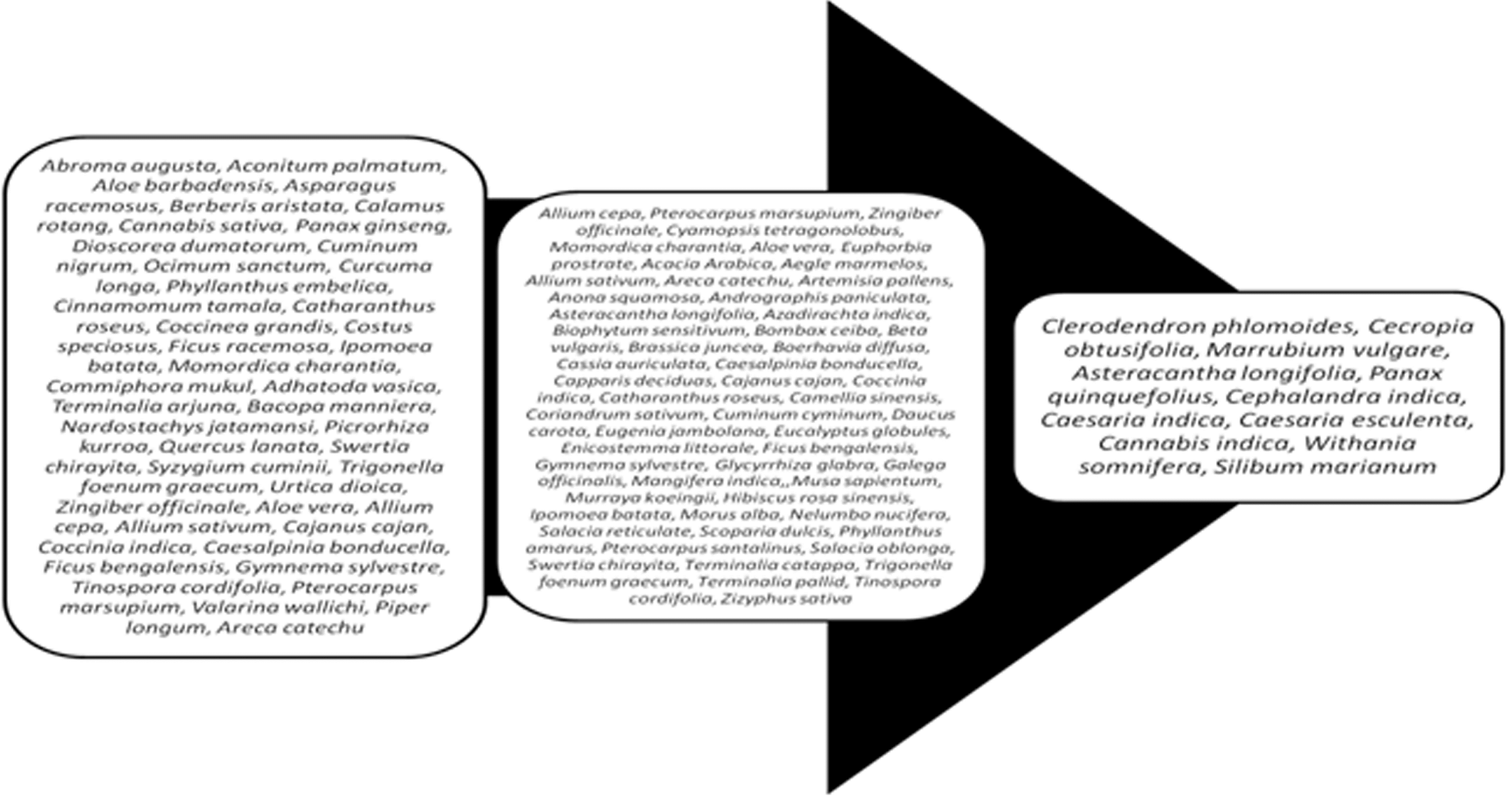

Supplement: Supplementary file 3 — Authors’ original file for figure 3 [file 40200_2013_71_MOESM3_ESM.tif]

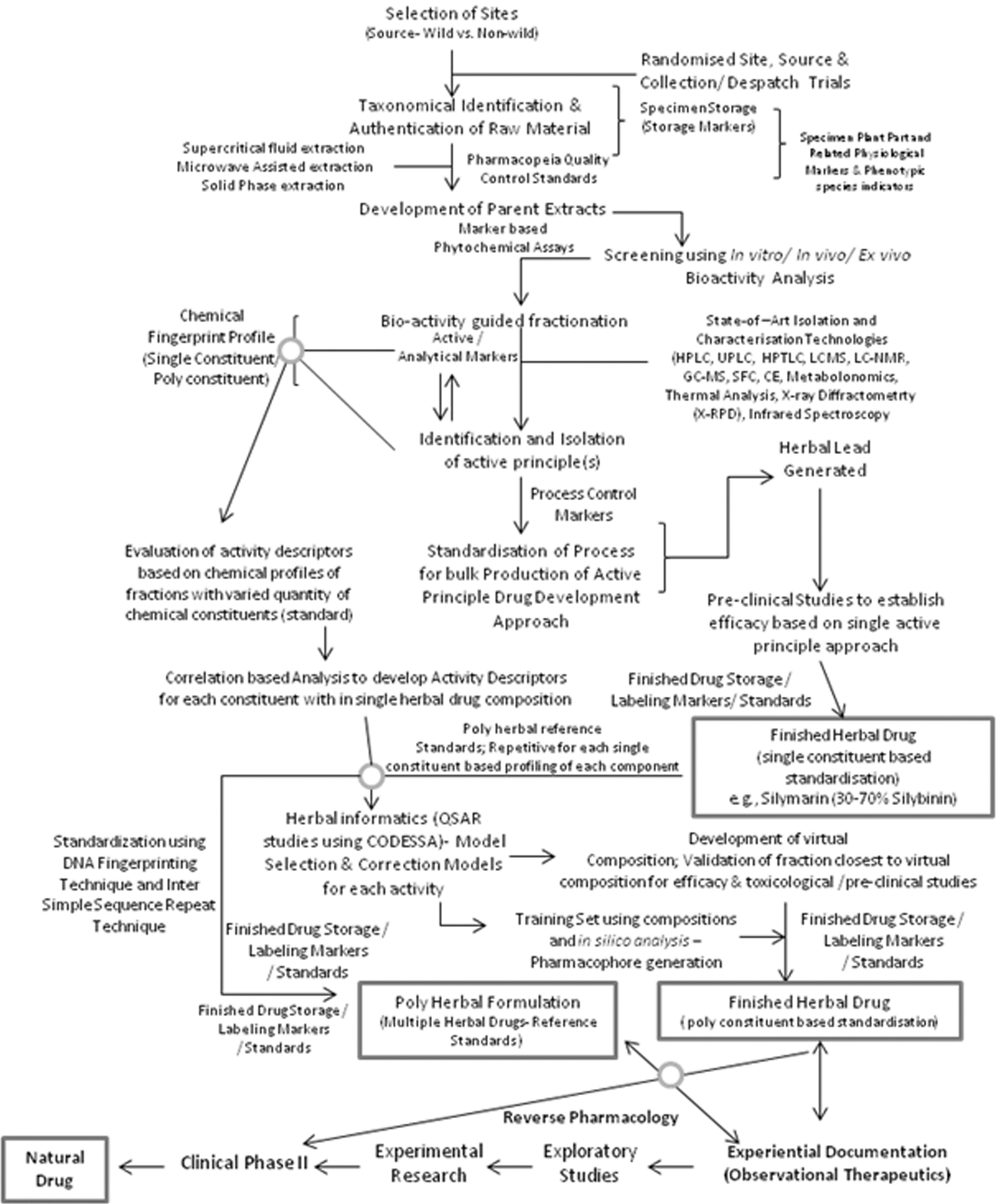

Supplement: Supplementary file 4 — Authors’ original file for figure 4 [file 40200_2013_71_MOESM4_ESM.tif]
